# Coccidian Infection Causes Oxidative Damage in Greenfinches

**DOI:** 10.1371/journal.pone.0036495

**Published:** 2012-05-15

**Authors:** Tuul Sepp, Ulvi Karu, Jonathan D. Blount, Elin Sild, Marju Männiste, Peeter Hõrak

**Affiliations:** 1 Department of Zoology, Institute of Ecology and Earth Sciences, The Centre of Excellence FIBIR, Tartu University, Tartu, Estonia; 2 Centre for Ecology and Conservation, College of Life and Environmental Sciences, University of Exeter, Penryn, United Kingdom; Arizona State University, United States of America

## Abstract

The main tenet of immunoecology is that individual variation in immune responsiveness is caused by the costs of immune responses to the hosts. Oxidative damage resulting from the excessive production of reactive oxygen species during immune response is hypothesized to form one of such costs. We tested this hypothesis in experimental coccidian infection model in greenfinches *Carduelis chloris.* Administration of isosporan coccidians to experimental birds did not affect indices of antioxidant protection (TAC and OXY), plasma triglyceride and carotenoid levels or body mass, indicating that pathological consequences of infection were generally mild. Infected birds had on average 8% higher levels of plasma malondialdehyde (MDA, a toxic end-product of lipid peroxidation) than un-infected birds. The birds that had highest MDA levels subsequent to experimental infection experienced the highest decrease in infection intensity. This observation is consistent with the idea that oxidative stress is a causative agent in the control of coccidiosis and supports the concept of oxidative costs of immune responses and parasite resistance. The finding that oxidative damage accompanies even the mild infection with a common parasite highlights the relevance of oxidative stress biology for the immunoecological research.

## Introduction

Parasites and pathogens are currently recognized as a major evolutionary force, responsible for the emergence of sophisticated defence mechanisms which eventually interfere with physiological and life history strategies of the hosts (reviewed by [1]). Although it might seem obvious that it would always be best to fight off parasites and diseases fast and efficiently, hosts still remain susceptible and immune responses vary widely between individuals. An ecological explanation for this apparent paradox is that immune responses are costly for the hosts. However, the question about the currencies used for paying the costs of activation of immune defences has remained poorly understood. The traditional view of animal ecologists has been that the costs involved in life-history trade-offs are basically energetic [2], which is in good agreement with the high metabolic burden of febrile acute phase responses (e.g., [3], [4], [5]). On the other hand, it has been also claimed that energetic demands required for maintenance of immune function and for mounting specific immune responses are negligible [6], [7]. Furthermore, experimental tests of these ideas have given contradictory results (reviewed by [8], [9], [10]). Therefore, an alternative hypothesis, proposing that costs of immune responses are primarily caused by the accompanying immunopathological tissue damages, is becoming increasingly popular [11], [12], [13], [14].

The vertebrate innate immune system protects the organism by producing reactive oxygen species in a process called oxidative burst. These oxygen species are highly reactive and destroy pathogens by damaging their proteins, lipids and DNA. These reactive species are not pathogen-specific and can also damage host tissues if there are not enough protective antioxidants present. Oxidative stress is a situation when the balance of pro-oxidants and antioxidants is shifted towards pro-oxidants and this causes oxidative damage to organisms’ own tissues [15]. Oxidative stress is believed to play an important role in senescence, expression of sexual ornamentation and sperm performance and can be linked with selection pressures to survival and reproduction [11], [16], [17], [18], [19]. Therefore, oxidative stress may appear to be one of the mechanisms that link immune function with life-history traits [14], [20]. Yet the evidence for this claim appears contradictory as a recent meta-analysis of avian studies showed only a weak association (4.1% of variance explained) between induced immune responses and markers of oxidative stress [20].

The aim of this study is to test whether experimental infection with common intestinal parasites – coccidians from the genus *Isospora* – generates oxidative damage in a passerine bird, the greenfinch. Coccidians are directly transmitted protozoans that cause massive production loss in poultry industry (e.g., [21], [22]) and affect fitness in wild birds (reviewed by [23], [24]). Inhabiting the intestinal epithelium, they directly inhibit the uptake of essential dietary components, including fat-soluble antioxidants such as vitamin E and carotenoids [24], [25]. However, the question of whether these fat-soluble antioxidants are efficient in prevention of oxidative damage *in vivo* is open [26], [27]. To our knowledge, only a single study of wild birds has addressed the question of whether coccidian infection affects oxidative balance in wild birds. Pap and colleagues [23] detected an increase in Total Antioxidant Status (TAS) in house sparrows (*Passer domesticus*) infected with isosporans as compared to medicated control birds. However, the interpretation of TAS is complicated [28], [29], not least because it does not assess the contribution of fat-soluble and enzymatic antioxidants [30], [31]. Here we use plasma malondialdehyde (MDA) levels for the assessment of potential oxidative damage accompanying coccidian infection. MDA is an end-product of peroxidative decomposition of unsaturated lipids; it is also mutagenic and cytotoxic and can damage membrane proteins [32] and is often considered as a presumptive marker of oxidative stress (e.g., [33]).

Several studies in domestic chickens have documented an increase in plasma MDA levels in response to coccidian infection [34], [35], [36], [37], [38]. However, all of those have relied on spectrophotometric detection of thiobarbituric acid reactive substances (TBARS) for the assessment of lipid peroxidation. This method has been severely criticized because of non-specificity and artefactual generation of TBARS during the assay (e.g., [32]). Quantification of MDA by high-performance liquid chromatography (HPLC) is devoid of such problems [39]. Furthermore, the coccidians of poultry belong to the different genus with higher pathogenicity (*Eimeria*) and the selection pressures on the immune function of domestic chicken are vastly different from those of the wild birds [40]. We were thus interested whether it would be possible to detect oxidative damage due to coccidian infection in a model species of immunoecological research by measurement of MDA by HPLC. Further, we asked whether higher individual plasma levels of MDA are associated with better resistance to infection. Such a hypothesis is based on oxidative destruction of Eimerian coccidians in poultry [22], [41]. For instance, it has been reported that exogenous nitric oxide is toxic to sporulated coccidian oocysts (reviewed by [35], [42]) and dietary additives that can generate oxidative stress are detrimental to the parasite development [22]. In order to monitor antioxidant defences of greenfinches, we tested whether coccidian infection induces changes in plasma antioxidant potential by measuring changes in two corresponding markers – TAC and OXY. TAC (Total Antioxidant Capacity) assay measures the capability of antioxidants in plasma to reduce the synthetic ABTS^+^ radical. OXY (Oxygen Radical Absorbance) assay quantifies the ability of the plasma antioxidants to withstand the oxidant action of hypochlorous acid. At least in greenfinches, the levels of TAC and OXY did not correlate, indicating that they represent different components of plasma antioxidativity [43]. On the basis of the results obtained in house sparrows [23], we predicted that our measurements of plasma antioxidant activity will increase in response to infection due to compensatory up-regulation of antioxidant protection systems in response to immune activation (e.g., [44]) or oxidative damages inflicted [29]. To assess the general physiological and nutritional impact of infection, we measured body mass, plasma triglycerides and carotenoids which are all known to decrease in response to coccidiosis-induced malabsorption of nutrients. Hence we predicted that all these parameters will decrease in infected birds. Furthermore, plasma carotenoids in greenfinches are directly correlated to those sequestered in feathers during moult [45], and thus, to carotenoid-based signals [46], [47]. We consider understanding the relationships between infection, antioxidant defences, oxidative damage and carotenoids important because coccidiosis of wild birds is becoming an increasingly popular model of parasite-mediated selection, particularly in the context of the interest of animal ecologists in carotenoid-based ornaments and oxidative stress ecology.

## Methods

### Study Protocol and Infection

Fifty six male wild greenfinches were captured in mist-nets at bird feeders in a garden in the city of Tartu (58° 22′ N; 26° 43′ E) on 28 and 29 December 2010. The birds were housed indoors in individual cages (27×51×55 cm) with sand-covered floors. Average temperature in the aviary during the experiment was 13.5±1.7 (SD) °C and average humidity was 43.1±4.9 (SD) %. The birds were supplied *ad libitum* with sunflower seeds and tap water. Birds were held on the natural day-length cycle on artificial lighting by luminophore tubes (Mazdafluor Prestiflux Brilliant 840, Dijon, France). The birds were released into their natural habitat on February 24. The study was conducted under the license from the Estonian Ministry of the Environment and the experiments comply with the current laws of the Estonian Republic.

Isosporan infection intensities were determined from faecal samples collected in the afternoon (two hours before the lights switched off) as described previously [24], [48]. Excreted oocysts for infecting experimental birds were collected from all the male birds during ten days before the start of anticoccidian medication and additionally from 22 female captive greenfinches during the 5-day period. Faecal samples were maintained in 2% potassium dichromate (K_2_Cr_2_O_7_) solution at room temperature and aerated daily. Inocula of sporulated oocysts for experimentally infecting were prepared as a single stock from all donor individuals as previously described [24], [48].

**Figure 1:**
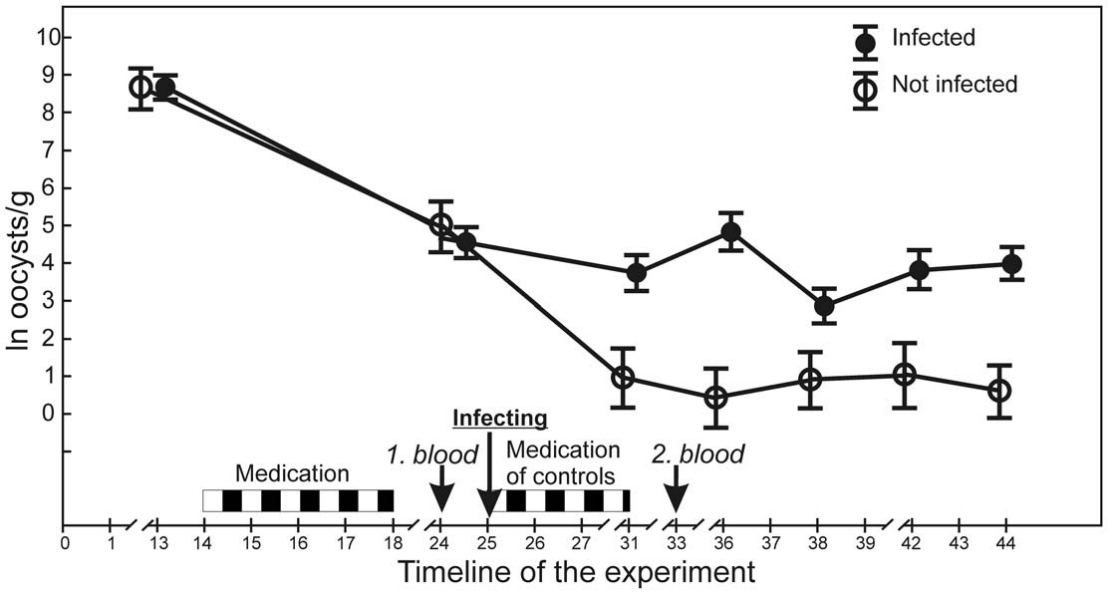
Timeline of the experiment and dynamic of the infection. Day 1 = 4 January. Effect of treatment: F_1,54_ = 13.2, P = 0.006; Effect of time: F_6,324_ = 52.9, P<0.00001; Effect of time*treatment F_6,324_ = 36.3, P<0.00001. Average infection intensities did not differ between infected and not infected birds before infecting (P = 0.6–1), while after infecting, infected group had significantly higher oocyst shedding in any date of measurement (P = 0.03–0.0001).

The time course of the experiment is shown in the Fig. 1. The birds were divided into a control group (16 individuals) and an infection group (40 individuals) on the basis of similar age composition, body mass and infection status. (The infection group was larger because we were interested in measurement of the covariation between hematological parameters and resistance to experimental infection.) 31% (5 individuals) in the control and 37.5% (15 individuals) in the experimental group were yearlings while the rest were older. Age of birds did not affect any of the studied parameters (all P-values >0.2). Average body mass, measured on the evening of 3 January was 31.8±2.7 (SD) g in the control and 31.3±2.3 (SD) g in the infection group (t = 0.64, P = 0.53). Average infection intensity, assessed on 16 and 17 January (i.e., before medication) was 41 231±90 983 (SD) oocysts per gram of faeces in the control group and 37 166±77 161 (SD) oocysts/g in the experimental group (z = −0.15, P = 0.88 in U-test).

All birds appeared infected with isosporan coccidians on 16 and 17 January. From 17 to 21 January all of the birds were subjected to five-day anticoccidian treatment with Intracox Oral (Interchemie, Castenary, The Netherlands) in order to standardize their infection status. The birds received 2 ml/L of the solution containing 25 mg/L Toltrazuril in their drinking water. The medication period was followed by a six-day recovery period as to enable excretion of the residues of the drug in order to make the birds susceptible to experimental infection.

From 28 January to 3 February, birds in the control group were subjected to another, 6-day medication period in order to increase differences in infection intensity between control and infected birds. (We were concerned about inefficient eradication of the infection by the single bout of medication; see results and Fig. 1.) In the evening of 28 January a dose of 2000 sporulated oocysts, diluted in 100 µL tap water was administered orally by micropipette to birds in the infection group. At the same time, the control group received the same amount of water. Faecal samples for monitoring the subsequent course of infection were collected on 3 January and 8, 10, 12 14, and 16 February.

Birds were first blood-sampled on 27 January, i.e., after all the birds had first received the 5-day period of medication and 5-day period of recovery from medication. A second blood sample was collected on 5 February, i.e., during the expected peak phase of the new infection in the infected group. By that time, 8 days had elapsed from the experimental infection in the infection group while the control group had received a second, 6-day period of medication and a two-day period of recovery from medication. The total amount of blood extracted each time was ca 200 µL and it was collected into 200 µL Microvette® tubes (K3E) with EDTA tripotassium salt as an anticoagulant. Blood sampling took place in the mornings before the lights turned on. Immediately after blood collection, tubes were placed into a cooled and light-protected box on snow and centrifuged within one hour from sampling for 10 min at 5000 g at 4°C to separate plasma from erythrocytes. Plasma was stored at –80°C until analyzed within two months. From 4 January (day 1 in the Fig. 1), all the birds started receiving 10 µg/mL carotenoid solution in their drinking water to compensate for naturally low carotenoid content of sunflower seeds. Such dose results in plasma carotenoid levels that are characteristic to wild greenfinches in winter (Table 1, [48], [49]). Carotenoid supplementation consisted of lutein and zeaxanthin (20:1, w/w), prepared from OroGlo liquid solution of 11 g/kg xanthophyll activity (Kemin AgriFoods Europe, Herentals, Belgium). Carotenoid supplementation lasted until 14 February and was temporally ceased from 17–21 January and 27 January –3 February during the periods of anticoccidian medication in order to avoid possible interference of Toltrazuril and carotenoids in the drinking water.

**Table 1:** Biochemical indices and body mass of greenfinches at first and second blood sampling (before and after experimental infection).

Variable	Gro p	Mean	SD	N
MDA (µM)	Control before infection	2.11	0.51	14
	Control after infection	1.92	0.29	15
	Infected before infection	2.13	0.50	34
	Infected after infection	2.20	0.28	34
				
OXY mM	Control before infection	195	67	14
	Control after infection	54	20	15
	Infected before infection	202	58	35
	Infected after infection	161	29	33
				
TAC mM	Control before infection	0.14	0.17	13
	Control after infection	0.14	0.15	13
	Infected before infection	0.15	0.19	35
	Infected after infection	0.13	0.18	31
				
Carotenoids (µg/mL)	Control before infection	9.7	6.9	13
	Control after infection	11.1	7.1	13
	Infected before infection	10.9	4.7	24
	Infected after infection	10.8	4.5	34
				
Triglycerides (g/L)	Control before infection	167	34	14
	Control after infection	178	37	13
	Infected before infection	180	35	30
	Infected after infection	174	41	31
				
Mass (g)	Control before infection	31.4	3.2	16
	Control after infection	29.6	2.5	16
	Infected before infection	31.4	2.6	40
	Infected after infection	29.2	2.5	40

### Biochemical Analyses

Concentration of carotenoids was determined spectrophotometrically from 15 µl of plasma, diluted in acetone as described by Sild *et al.* [50], using lutein (Sigma X 6250) as a standard. Plasma triglyceride concentration was determined from 2.5 µL samples by the GPO-PAP method (Human GmbH, Wiesbaden, Germany). Plasma Total Antioxidant Capacity (TAC) was measured from 5 µL plasma samples according to the method described by Erel [51] with minor modifications as described by Sepp *et al.* [49]. The assay is based on the capacity of antioxidants in the solution to decolorize the ABTS^+^ (2, 2– azinobis (3-ethylbenzothiazoline-6-sulfonate) according to their concentrations and antioxidant capacities. Main contributors to TAC are plasma uric acid and free sulfhydryl groups of proteins [49], [51]. The results are quantified in mM Trolox (water soluble vitamin E analogue) equivalents. Plasma Oxygen Radical Absorbance (OXY) was measured with a OXY-adsorbent test (Diacron International, Grosseto, Italy) from 5 µL plasma samples according to the manufacturers instructions (see [52]). This test quantifies the ability of the plasma non-enzymatic antioxidant compounds to cope with the *in vitro* oxidant action of hypochlorous acid (HOCl; an endogenously produced oxidant). OXY values were not correlated with plasma uric acid content in zebra finches [52] and greenfinches [43] and among the birds sampled in the current experiment, plasma levels of TAC did not correlated with those of OXY or uric acid [43]. The concentrations of OXY are expressed as mM of HOCl neutralized. The repeatability [53] of OXY was 0.79 (F_9,10_ = 8.3, P = 0.0007). Plasma concentrations of MDA were assayed using HPLC as described previously [54], [55], except that we used 10 µL of plasma and thus volumes of all reagents in the incubation mixture were adjusted proportionately (i.e. 10 µl butylated hydroxytoluene solution; 80 µl phosphoric acid solution; 20 µl thiobarbituric acid solution). The repeatability of plasma MDA was 0.86 (F_17,18_ = 13.3, P<0.00001).

### Statistics

The effects of experimental infection on the dynamics of coccidiosis were tested using repeated measures ANOVA on ln-transformed values of infection intensity. Effects of treatments on changes in the values of other parameters between the first and second blood sampling were tested in ANCOVAs adjusting for the initial trait values (Table 2). Assumptions for parametric models (normality of residuals, homogeneity of variances) were met for all the models. All tests are two-tailed with an α-level below 0.05 as a criterion for significance. Sample sizes vary between different measures due to our inability to collect sufficient amount of plasma from all the birds.

**Table 2:** Effects of experimental coccidian infection on changes of body mass and biochemical parameters of greenfinches between first and second blood sampling.

Dependent variable	Predictors	df	F	η^2^	P
MDA change	Initial value	1,39	87.8	0.69	<0.0001
	Infection	1,39	5.9	0.13	0.020
					
OXY change	Initial value	1,39	271.6	0.87	<0.0001
	Infection	1,39	1.1		0.293
					
TAC change	Initial value	1,33	16.1	0.33	0.0003
	Infection	1,33	0.1		0.752
					
Carotenoid change	Initial value	1,32	7.3	0.12	0.011
	Infection	1,32	0.9		0.340
					
Triglyceride change	Initial value	1,33	1.9		0.178
	Infection	1,33	1.6		0.220
					
Mass change	Initial value	1,53	14.4	0.21	0.0004
	Infection	1,53	0.6		0.454

η^2^ stands for coefficients of partial determination, describing the proportion of total variation attributable to the predictor variable, partialling out other factors from the total nonerror variation. Average trait values are presented in Table 1.

## Results

Anticoccidian treatment was not fully efficient in eradication of the infection because on 27 January (day 24 in Fig. 1) only 2 birds (12.5%) in the control group and 10 birds (25%) in infection group appeared parasite free. Average infection intensities after medication and recovery period, however, had dropped by more than 50 times as compared to before-medication period in mid-January and did not differ between groups ((375±498 (SD) oocysts/g in control group vs 806±2228 (SD) oocysts/g in experimental group (z = 0.27, P = 0.55 in U-test), see also Fig. 1). Subsequent to experimental infection, oocyst loads in the infected group remained significantly higher than in the un-infected group although the average infection intensity never reached the initial level observed before medication (Fig. 1).

**Figure 2:**
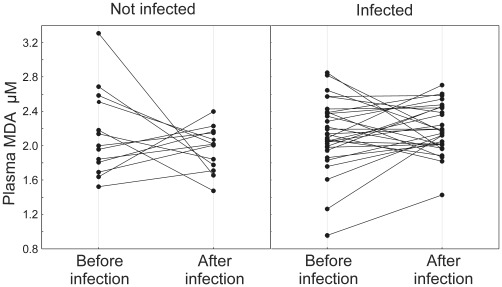
Effect of experimental coccidian infection on changes of plasma MDA levels between the first and second blood sampling. 29 infected and 13 un-infected birds. Average ± SD change = −0.18±0.66 µM for control birds and 0.05±0.40 µM in infected birds. See Table 2 for P-values.

Plasma MDA levels did not correlate significantly with infection intensities before experimental infection (r_s_ = 0.03–0.12, P = 0.8–0.4, n = 48). Plasma MDA levels did not differ between treatment groups before experimental infection (2.10±0.51 (SD) µM, n = 14 in un-infected group vs 2.12±0.50 (SD) µM, n = 34 in infected group; t = −0.15; P = 0.88), while at second blood sampling infected birds had on average 8.2% higher MDA levels than un-infected birds (1.92±0.29 (SD) µM, n = 15 in un-infected group vs 2.19±0.28 (SD) µM, n = 34 in infected group; t = −3.23; P = 0.002). The change in plasma MDA (Fig. 2) was also significant while changes in other biochemical measures and body mass did not differ between treatment groups (Table 2). Since plasma MDA was associated with experimental infection, we asked whether it also correlates with parasite resistance at individual level. This was indeed the case. The birds that had highest MDA levels at second blood sampling experienced the highest decrease in infection intensity by the end of the experiment as compared to pre-infection period (Table 3).

**Table 3:** Relationship between change in infection intensity between day 44 (last sampling of infection) and day 24 (before infecting) and plasma MDA levels at second blood sampling in an ANCOVA adjusting for a initial value of infection intensity and infection treatment. Interaction terms with infection treatment were not significant (P>0.9).

Predictors	df	F	η^2^	P	β±SE
Intensity of infection in day 24	1,45	34.6	0.43	<0.00001	−0.55±0.09
Plasma MDA after infecting	1,45	4.5	0.09	0.039	−0.21±0.10
Infection treatment	1,45	27.7	0.38	<0.00001	−0.54±0.10

## Discussion

Anticoccidian treatment did not result in total elimination of parasites from the faeces, which is comparable to earlier studies on greenfinches [24] and other passerines [56], [57]. However, the average pre-medication infection intensity was never reached in the experimentally infected group (Fig. 1). This result differs from our previous infection experiments with greenfinches [24], [58] where inoculation with the mixture of oocysts from different donors resulted in increase of infection intensity above the initial level. The most likely explanation for this discrepancy is that we used a novel coccidiostatic, Toltrazuril, in the current study. Unlike sulphonamide drugs, Toltrazuril binds to the intestinal mucosa, the site of action against endogenous stages of coccidians, where it resides longer than in body fluids [59], [60], Toltrazuril leads to a reduction of enzymes of the respiratory chain of the parasites and it is efficient against all intracellular developmental stages of Eimerian coccidians. At the same time, it does not interfere with development of natural immunity (reviewed by [61], [62]). Similarly to current study, Toltrazuril treatment prevented the rise of infection to pre-medication levels in an experimental infection of house sparrows [56]. Experiments aiming to obtain the maximum increase in Isosporan infection intensity should therefore avoid treatment of experimental birds with Toltrazuril before infecting. On the other hand, Toltrazuril should be considered superior to any sulphonamide drugs in studies of the health impact of coccidiosis because it specifically reacts on the apicoplast. Apicoplast is an organelle of botanical origin, which is unique to the apicomplexan parasites. Treatment of coccidiosis by Toltrazuril is thus devoid of any problems accompanying the use of broad spectrum antibiotics, such as incidentally affecting some bacterial infection that the experimenters are not able to monitor.

During the peak phase of infection, infected greenfinches had on average 8% higher levels of plasma MDA than un-infected birds. This difference was mainly due to 8.6% reduction of plasma MDA-levels in medicated group as compared to the pre-infection levels. In infected group, plasma MDA levels rose on average only 3.2%. This pattern suggests that the experimental effect on plasma MDA level was primarily caused by the medication treatment that reduced the amount of lipid peroxidation accompanying chronic, submaximal coccidian infection. On the basis of the dynamics of infection depicted in Fig.1, one would predict that the plasma MDA levels before anticoccidian treatment would have been even higher than during our first blood sampling event. Such possibility would be interesting to test in future studies on oxidative damages of coccidiosis in wild birds. If true, this would suggest that these intestinal parasites have a major impact on oxidative status of wild birds. Given the commonness of coccidiosis, it would also imply that birds can normally cope with oxidative consequences of this infection, e.g., by selective intake of dietary antioxidants [63].

Why was the infection associated with lipid peroxidation? We consider it unlikely that the effect was due to possible direct antioxidant effect of Toltrazuril *per se* for the following reasons. First, the terminal elimination half-life of Toltrazuril in the plasma of broilers was 11.4 hours [64], thus given that our control birds had not been receiving Toltrazuril for two days before blood sampling, it seems unlikely that they had any effective residues of the drug in their blood. Second, no significant differences were found in plasma MDA levels in uninfected chickens treated with Toltrazuril vs water [38]. Interestingly, the same study showed that Toltrazuril inhibited the activity of catalase, an enzyme catalyzing the decomposition of the highly reactive oxygen species hydrogen peroxide. So, if anything, one would expect the drug to increase oxidative damages rather than reducing them. Third, the different doses of Toltrazuril had no differential effect on several immune and physiological parameters of house sparrows [23]. We have thus good reasons to assume causal relationships between coccidian infection and increased lipid peroxidation. Theoretically, such a relationship could be caused either by direct action of coccidians or by the host responses to infection. However, there are no indications in support of the first possibility. On the contrary, reactive oxygen and nitrogen species are toxic to coccidia [65], so it would not be in the interest of parasites to initiate the processes leading to lipid peroxidation. We thus consider it most likely that higher plasma MDA levels in infected birds indicate oxidative damage generated during the immune response against Isosporan infection. For instance, Eimerian infection in domestic chickens elicits production of reactive oxygen species such as superoxide O_2_^−^ in activated macrophages, which either itself or by initiating free radical cascades causes lipid peroxidation [66], [67]. In addition, the infection of Eimerian coccidians in domestic chickens induces macrophages to produce nitric oxide, which is an important mechanism of parasite destruction [42] because NO and its reaction product with superoxide, the peroxynitrite (ONOO^−^), are toxic to parasites [65]. Importantly, peroxynitrite is also considered as one of the most important initiators of the oxidative damage [68]. Further, the result that infection did not reduce body mass, plasma triglycerides and carotenoids reinforces the conclusion that oxidative damage primarily resulted from the immune responses rather than from malabsorption of dietary antioxidants. To our knowledge, this is the first evidence of an association between coccidian infection and oxidative damage in a wild animal species. This result compares favourably with those obtained in experimental Eimerian infections in domestic chicken [34], [35], [36], [37], [38].

Lack of an effect of experimental infection on body mass, plasma triglycerides and carotenoids differs from findings of previous studies [24], [58] where the greenfinches were treated with sulphonamide antibiotics before infection. This discrepancy can be most likely explained by the lasting effects of pre-experimental treatment with Toltrazuril in the infected birds in the current study, as infection intensities never raised to pre-medication level. Thus, our experiment enabled us to establish that even infection with a common parasite with generally mild pathological consequences induced oxidative damage in the host. Furthermore, plasma MDA concentration appeared the single (and hence most sensitive) biomarker amongst the studied variables. This reinforces the contention of the potential importance of oxidative stress in anti-parasite defences [14]. On the other hand, our results also predict that infection experiments on immunologically naïve or non-medicated animals that increase the intensity of coccidiosis above pre-infection levels should detect even higher increases in oxidative damage due to higher immunogenicity. For instance, experiments in poultry that have detected the diverse physiological effects of coccidiosis have been always conducted on immunologically naïve chicks (reviewed by [22]). Presumably, such experiments would appear useful for elucidation of the physiological impact of oxidative stress in ecological model species. Obtaining naturally un-infected wild birds for such experiments, however, might constrain the selection of species, as for instance, all the greenfinches we have used in our experiments have been naturally infected.

The final interesting result of this study was that birds with highest plasma MDA levels obtained the highest proportional decline in infection intensity by the end of the study. Because oxidative stress is often implicated as causative agent of the control of Eimerian infection in domestic chickens (e.g., [41]), it might be tempting to speculate that similar mechanisms act also in Isosporan infection. Such an interpretation would fit into the general framework of oxidative costs of immune responses and parasite resistance [13], [14], [20]. However, to demonstrate the ecological relevance of this concept, one needs to prove that increased lipid peroxidation levels in more parasite-resistant individuals actually indicate accrual of pathological damage that would normally impinge on any components of fitness.

In conclusion, our results are encouraging with respect to the potential utility of Isosporan coccidiosis as a model system for investigation of the nexus between immune function and oxidative stress biology. Coccidians are the most prevalent avian parasites [69] with well established pathological effects (reviewed by [23]), so the ecological relevance of this kind of research is obvious. The main result of the current study – that plasma MDA was the single biochemical variable that responded to experimental infection with generally mild consequences – is in accordance with the relevance of the potential immunopathological impact of oxidative stress in an ecological context. We predict that more such connections will be found in the forthcoming years because the proper and precise measurement of lipid peroxidation has only recently reached ecological studies of wild animals, and the studies that have used such methods have indeed revealed ecologically relevant patterns (e.g., [54], [55], [70], [71], [72]). Further studies in this area would benefit from applying even more diverse assays for assessment of damage generated by the reactive species, such as oxidative damage to DNA (e.g., [73], [74]) and proteins [75]. Ultimately, the associations between indices of oxidative damage and different components of fitness need to be assessed in field studies.
